# Relationship Between Electronic Device Usage and Frailty Among Older Adults Living Alone in South Korea: Analysis of the Mediating Effect of Nutrition Management

**DOI:** 10.3390/healthcare14060816

**Published:** 2026-03-23

**Authors:** Kawoun Seo

**Affiliations:** Department of Nursing, Joongbu University, Geumsan-gun 32713, Republic of Korea; kwseo@joongbu.ac.kr

**Keywords:** big data, elderly, electronic device, frailty, nutrition

## Abstract

**Background/Objective:** Frailty is a major public health concern among older adults, particularly those living alone who may experience limited social support and increased nutritional vulnerability. With the rapid expansion of digital technologies, electronic device usage has become an important factor influencing daily life and health behaviors in older populations. This study aimed to examine the association between electronic device usage and frailty among older adults living alone and to investigate whether nutrition management mediates this relationship. **Methods:** This cross-sectional study conducted a secondary analysis using data from the 2023 Korean Elderly Survey. A total of 3423 adults aged ≥ 65 years who were living alone and completed the survey independently were included in the analysis. Descriptive statistics, independent *t*-tests, one-way analysis of variance, and Pearson correlation analyses were performed using SPSS version 26.0. Mediation analysis was conducted using Model 4 of the PROCESS macro (version 4.1) to examine the mediating role of nutrition management in the relationship between electronic device usage and frailty. **Results:** Electronic device usage was negatively associated with frailty and positively associated with nutrition management. Frailty was significantly higher among individuals with poorer nutrition management. Mediation analysis indicated that electronic device usage had both a direct effect on frailty and an indirect effect through nutrition management. Nutrition management partially mediated the relationship between electronic device usage and frailty, accounting for approximately 18% of the total effect. **Conclusions:** Electronic device usage was associated with lower levels of frailty among older adults living alone, and this relationship was partially mediated by nutrition management. These findings suggest that improving digital engagement may support better nutrition management and potentially contribute to frailty prevention among older adults living alone. Future longitudinal studies are needed to clarify the causal relationships among these variables.

## 1. Introduction

Frailty is a state of increased vulnerability in which individuals exhibit a reduced ability to cope with stressors originating from aging or chronic disease [1]. Fried et al. defined frailty using the following five criteria: unintentional weight loss, exhaustion, low physical activity, slowed walking speed, and reduced grip strength [1]. Frailty is a major risk factor for adverse health outcomes in older adults, as it reflects a decline in physical resilience that increases susceptibility to illness. In particular, frailty is associated with a higher risk of falls, injuries, physical disability, and hospitalization, and when left unmanaged, it may ultimately result in mortality in older populations [1,2,3,4]. Importantly, frailty is a preventable and potentially reversible condition. Unlike established disabilities, early detection and appropriate management can restore individuals to a healthier state. Therefore, early prevention and intervention strategies for frailty are required.

Frailty results from the interaction of multiple factors. Previous studies have indicated that older adults living alone are particularly vulnerable to frailty due to reduced social support and limited access to essential daily resources [5]. In Korea, where the population is aging rapidly, individuals aged ≥ 65 years accounted for 19.2% of the total population in 2024, with 37.8% of this group residing in single-person households. This equates to approximately one in four people in the overall population and one in three older adults [6]. Frailty not only adversely effects the health and quality of life of older adults but also imposes substantial human and economic burdens related to their care at both family and national levels. Because frailty can compromise the ability of older adults living alone to maintain independence, there exists a need for targeted efforts to prevent and delay its onset in this population.

Older adults living alone frequently face challenges such as limited access to health care, social isolation, and heightened nutritional vulnerability [7]. In particular, they are at an elevated risk of malnutrition due to inadequate dietary intake [8]. Poor nutrition among this population may result from financial constraints, social isolation, or physical and health-related limitations [9]. For example, difficulties with walking or driving can hinder grocery shopping or dining out. Moreover, the recent expansion of online ordering platforms and the transition to electronic kiosks for ordering and payment in restaurants have introduced additional barriers for older adults who have limited familiarity with digital technologies. Paradoxically, however, these same technology-based systems may also offer potential solutions to alleviate the challenges faced by older adults living alone.

The Fourth Industrial Revolution and the expansion of the internet-based information society have made electronic devices an integral component of daily life [10]. During the COVID-19 pandemic, non-face-to-face lifestyles that depend on electronic devices increased as a consequence of social distancing measures. Therefore, the ability to use electronic devices has become a fundamental skill for older adults in managing everyday activities [11]. It has also been demonstrated that electronic device usage among older adults promotes healthy aging and helps reduce loneliness [12]. In particular, access to online services through electronic devices may provide an effective solution for malnutrition among older adults living alone, who often experience social isolation and limited access to in-person support. For instance, in Korea, groceries can be ordered via mobile applications and delivered fresh to the home the following morning, and prepared meals can be selected and consumed based on photographic information. Accordingly, improved electronic device usage among older adults living alone may improve nutrition management and help prevent frailty.

Among the various pathways linking digital engagement to frailty, nutrition management may constitute a particularly critical mechanism. Malnutrition is one of the most significant modifiable risk factors for frailty, as inadequate dietary intake accelerates muscle loss, functional decline, and overall vulnerability in older adults [8,13]. Older adults living alone are especially vulnerable to nutritional risk due to limited meal preparation, diminished appetite, and barriers to accessing food [14]. In this context, electronic devices may play a crucial role in facilitating food acquisition, nutrition-related information seeking, and access to meal delivery services. Thus, nutrition management may serve as a key pathway through which electronic device usage influences frailty outcomes. Therefore, in this study, data from the Korean Elderly Survey of individuals aged ≥ 65 years were analyzed to examine the impact of electronic device usage on frailty among older adults living alone, with a particular focus on nutrition management as a mediating factor. Based on the existing literature, this study addresses the following research questions:

1. Is electronic device use associated with frailty among older adults living alone?
2. Is electronic device use associated with nutrition management?
3. Does nutrition management mediate the relationship between electronic device use and frailty?

## 2. Materials and Methods

### 2.1. Study Design

This cross-sectional study examined the mediating effect of nutrition management on the relationship between electronic device usage and frailty in older adults living alone.

### 2.2. Data Source and Participants

This study is a secondary analysis of data from the 2023 Korean Elderly Survey, which is conducted every 3 years by the Ministry of Health and Welfare of the Republic of Korea according to the Elderly Welfare Act. The survey aims to determine the overall health status and living conditions of older adults. Raw data were obtained through the Health and Welfare Data Portal after the approval of a research proposal and adherence to established procedures. Data collection was accomplished through face-to-face interviews conducted by trained investigators using a structured questionnaire developed by the research team. The survey was administered between 4 September and 12 November 2023, targeting adults aged ≥ 65 years across 977 districts nationwide. A total of 10,078 individuals participated in the survey. To evaluate frailty among older adults living alone, the following inclusion criteria were applied: (1) individuals aged ≥ 65 years, (2) individuals living in single-person households, and (3) individuals who completed the survey themselves. Exclusion criteria were (1) individuals living in households with two or more members and (2) individuals who provided proxy responses. Among the 10,078 respondents, 3423 reported living alone. Therefore, the final analysis sample comprised these 3423 participants.

### 2.3. Measures

This study examined six general characteristics, 13 questions on digital literacy, and 10 questions on nutrition management. General characteristics included age, gender, education level, occupation, subjective health status, and sleep quality.

#### 2.3.1. Electronic Device Usage

Electronic device usage was defined as the ability to access and use digital information through electronic devices such as smartphones, desktop computers, and laptops. Based on the framework proposed by Koo et al. [15], this study used items from the 2023 Survey on the Status of the Elderly that evaluated engagement in various digital activities. These activities included receiving and sending messages (e.g., text messages, KakaoTalk, and Telegram), making video calls, searching for information (e.g., news and weather), taking photos or videos, listening to music (e.g., MP3s and radio), playing games, watching videos (e.g., movies, television programs, and YouTube), using social networking services (e.g., blogs, online communities, Band, Twitter, Facebook, and Instagram), engaging in e-commerce (e.g., online shopping, reservations, and bookings), conducting financial transactions (e.g., internet banking and securities trading), searching for and installing applications, and using automated machines (kiosks) for ordering or applications in settings such as restaurants and hospitals. A total of 13 items were included. Each item was coded as 1 if the activity was used and 0 if not used, yielding a total score ranging from 0 to 13, where higher scores indicated greater levels of digital information literacy.

#### 2.3.2. Frailty

Frailty was evaluated using the K-FRAIL scale, which provides a frailty score based on five components: fatigue, resistance, ambulation, illness, and weight loss. This tool was originally developed by Morley et al. [16] and subsequently modified into a Korean version by Jung et al. [17]. This tool contains five items, viz., fatigue, resistance, mobility, chronic illness, and weight loss. Each item is given 1 point if it is true and 0 points if it is not true. The total score ranges from 0 to 5, with higher scores indicating more severe frailty.

#### 2.3.3. Nutrition Management

Nutrition management was measured using the Determine Your Nutrition Health checklist developed by Posner et al. [18], which was validated in Korea by Moon and Kong [19]. The DETERMINE checklist is widely used as a screening tool to identify nutritional risk among older adults rather than as a direct measure of nutrition management behaviors. This tool contains 10 items, each with a “yes” or “no” response. In this study, higher scores were defined as indicating greater nutritional risk, which corresponds to poorer nutrition management. On a 10-point scale, scores of 0–2 indicate good nutrition management, scores of 3–5 indicate caution in nutrition management, and scores of ≥6 indicate nutrition management requiring improvement.

### 2.4. Statistical Analysis

Statistical analyses were conducted using SPSS (version 26.0; IBM Corp., Armonk, NY, USA) and the PROCESS Macro for SPSS (version 4.1). Descriptive statistics were used to summarize the general characteristics of the participants and the study variables. Independent *t*-tests and one-way analysis of variance were conducted to examine differences in frailty according to general characteristics. Pearson correlation coefficients were calculated to evaluate the relationships among electronic device usage, nutrition management, and frailty. To examine whether nutrition management mediated the relationship between electronic device usage and frailty, a mediation analysis was performed using Model 4 of PROCESS Macro (version 4.1). The significance of the indirect effect was tested using bootstrapping with 5000 resamples, and the statistical significance was determined based on a 95% confidence interval. A *p*-value of <0.05 was considered statistically significant.

## 3. Results

### 3.1. General Characteristics and Study Variables

The mean age of the participants was 75.57 years (range: 65–98 years). The majority of them were women (77.6%), and more than half (57.3%) had an elementary school education or less. Only 37.2% of them were employed, and 32.6% reported perceiving themselves as healthy. Regarding sleep quality, 42.3% reported good sleep, whereas 22.2% reported poor sleep.

Frailty differed significantly according to gender, educational attainment, employment status, perceived health status, and quality of sleep. Women showed higher frailty scores than men, and individuals with lower education levels exhibited greater frailty. Furthermore, unemployment, poorer self-rated health, and lower sleep quality were associated with increased frailty. The mean score for nutrition management was 3.12 ± 3.23, and the mean score for electronic device usage was 3.42 ± 3.41. The mean frailty score was 0.81 ± 1.01 (Table 1).

### 3.2. Correlations Between Study Variables

Frailty correlated negatively with electronic device usage and positively with nutrition management. In other words, frailty was less prevalent with greater electronic device usage and more prevalent with poor nutrition management. Moreover, a negative correlation was detected between electronic device usage and nutrition management. In other words, greater electronic device usage was associated with better nutrition management (Table 2).

### 3.3. Mediating Effect of Nutrition Management on the Relationship Between Electronic Device Usage and Frailty

**Figure 1:**
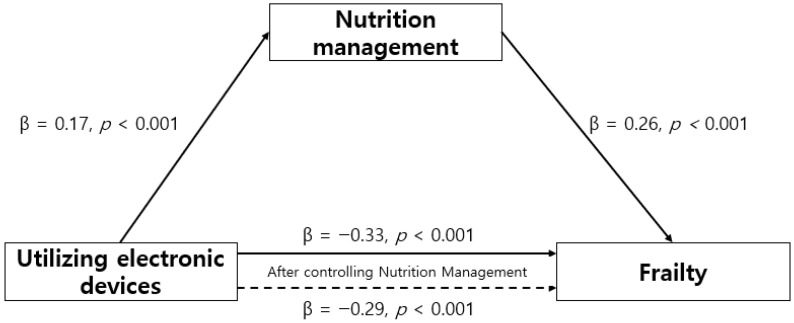
Mediating effect of variables.

The mediating effect of nutrition management on the relationship between electronic device usage and frailty among older adults living alone was evaluated using a mediation analysis conducted using Model 4 of PROCESS Macro. The detailed results of this analysis are shown in Table 3. In Step 1, electronic device usage exerted a statistically significant effect on nutrition management (β = −0.17, *p* < 0.001). In Step 2, electronic device usage exerted a significant direct effect on frailty (β = −0.33, *p* < 0.001). In Step 3, when both electronic device usage and nutrition management were included in the model, both variables significantly affected frailty (β = −0.29, *p* < 0.001; β = 0.26, *p* < 0.001). The reduction in the standardized coefficient for electronic device usage from Step 2 to Step 3 indicates that nutrition management partially mediated the relationship between electronic device usage and frailty. Hence, electronic device usage affected frailty both directly and indirectly through nutrition management, and the mediating effect accounted for 18.0% of the total explanatory power (Table 3) (Figure 1). Bootstrapping analysis further confirmed the significance of the indirect effect, with an effect size of −0.05 and a 95% confidence interval ranging from −0.06 to −0.04. As the confidence interval did not include zero, the mediating effect was statistically significant (Table 4).

## 4. Discussion

This study investigated electronic device usage, frailty, and nutrition management among older adults living alone using data from the Korean Elderly Survey. The aim was also to identify the mediating role of nutrition management in the relationship between electronic device usage and frailty.

The mean electronic device usage score of the participants was 3.42 of 13, which is comparable to the range of 0–3 of 9 reported by Koo et al. [15]. Moreover, among the various digital activities, the most commonly used functions were messaging, video watching, and information searching, whereas more complex activities, such as financial transactions and application installation, were less frequently used. These findings suggest that relatively simple digital functions play a vital role in the daily lives of older adults living alone. Previous research indicates that prolonged television viewing or computer use among older adults increases sedentary behavior and may adversely impact health. Conversely, mobile devices allow users to remain physically mobile during use and provide convenient access to health- and well-being-related information, which has been demonstrated to exert positive health effects [20]. The findings of the present study are consistent with this evidence. Remarkably, the electronic device usage measures used in this study comprised activities such as information searching, electronic commerce, and ordering through applications or automated systems, extending beyond passive forms of device use, such as watching television and playing computer games. Because older adults living alone are particularly vulnerable to limited social support, improved electronic device literacy may help reduce barriers to information access and increase physical functioning and access to social services. Furthermore, higher levels of electronic device literacy among older adults have been associated with reduced depression [21]. Accordingly, there exists a need to develop programs aimed at improving electronic device usage skills among older adults living alone.

The nutritional status score of older adults living alone in Korea was 3.12 points, which is within the “cautionary” range according to the evaluation criteria of the measurement tool [18]. This score is lesser than the 4.84 points reported in a study of older adults in Thailand by Harnirattisa et al. [22], suggesting that Korean older adults living alone have a relatively better nutritional status. The differences observed between the studies may be influenced by cultural values and social changes related to elder care in Thailand [22], a context that differs in several respects from the Korean society. In Korea, rapid population aging has resulted in an increase in both the absolute number of older adults and the proportion of those living alone [7]. Consequently, the number of older adults supported per working-age adult has increased, thereby elevating the individual and societal burden of elder care [23]. In response, the Korean government has implemented various older adult care initiatives. For instance, under the Community Integrated Health Promotion Project, older adults are provided with nutrition education, healthy eating programs, and healthy snack or lunch box services, which are often related to other support programs. Nevertheless, considering the physical decline and social isolation commonly experienced by older adults living alone, there exists a need for systematic nutrition monitoring and tailored nutrition management interventions that specifically address their circumstances. Hence, the development of an integrated nutrition management model targeting older adults living alone is recommended.

In the present study, the mean frailty score was 0.81, which is within the normal range on a 5-point scale. Frailty levels differed significantly according to gender, education level, employment status, subjective health status, and sleep quality. These results are consistent with those of previous studies on frailty among older adults living alone [7,8]. The higher prevalence of frailty observed in women may be attributed to their longer life expectancy, because advancing age is closely associated with increased frailty [8]. Although findings concerning education level have been inconsistent, several studies report a lower prevalence of frailty among individuals with higher educational attainment. This association may be explained by social factors, such as improved access to health-related information and higher socioeconomic status among individuals with more education [24]. Therefore, future studies should use path analyses to explore the multifactorial effects on frailty, including education level, economic status, and access to health care. Remarkably, several previous studies have evaluated frailty by classifying participants into normal, pre-frail, and frail groups rather than reporting mean frailty scores. Although estimating the prevalence of frailty or pre-frailty within specific groups is informative, presenting average frailty scores would allow for a more nuanced comparison of frailty severity across groups and improve the interpretation of group differences.

In the present study, results showed that electronic device literacy exerted a direct effect on frailty among older adults living alone and an indirect effect through nutrition management. The use of electronic devices among older adults remains a subject of debate. On the one hand, the use of nonmobile devices, such as televisions and desktop computers, has been associated with increased sedentary behavior, which may adversely impact health [20]. On the other hand, higher levels of digital literacy among older adults improve access to health-related information and medical services, thereby contributing to improved physical and mental well-being [25]. In Korea, the widespread availability of mobile app–based delivery services enables older adults, including those with physical limitations, to receive fresh food ingredients and warm meals at home. This environment suggests that greater electronic device literacy facilitates better nutrition management and in turn reduces frailty. Therefore, there exists a need for educational programs aimed at improving electronic device literacy among older adults living alone. Furthermore, it is essential to consider the integration of digital device–based interventions when designing and implementing nutrition education programs for this population. Individuals who are less frail or cognitively healthier also more probably adopt and use digital technologies. Previous research has suggested that digital technology adoption among older adults partially reflects their underlying functional capacity and cognitive ability [26]. Moreover, longitudinal research has suggested that computer use influences and reflects cognitive, social, and health-related functioning over time [27]. Therefore, future longitudinal studies are required to clarify the temporal dynamics of these relationships. In addition, several unmeasured factors may simultaneously affect both digital engagement and nutritional status. Socioeconomic status, chronic health conditions, cognitive functioning, and social support may simultaneously influence the ability to use electronic devices and maintain adequate nutrition. Future studies should incorporate these factors to better clarify the mechanisms underlying these associations.

This study has several limitations. First, as it was conducted from a cross-sectional perspective, there exist limitations in establishing clear causal relationships. Another limitation is that individuals who required proxy responses were excluded from this analysis. These individuals are likely to be older, frailer, or cognitively impaired and may exhibit lower levels of digital engagement and greater nutritional vulnerability. Hence, the findings may underestimate the relationship among digital engagement, nutrition management, and frailty. Despite these limitations in determining the causal relationships among variables, the large sample size enabled us to confirm that electronic device usage among older adults living alone influences frailty through nutrition management. These findings indicate the need to provide educational interventions aimed at improving electronic device usage among socially isolated older adults with physical limitations. Furthermore, incorporating electronic device usage into nutrition management programs may help reduce frailty in this population.

## 5. Conclusions

This study examined data from the Korean Elderly Survey to investigate the associations among electronic device use, frailty, and nutritional management in older adults living alone. It further explored whether nutritional management mediates the relationship between electronic device usage and frailty. Results showed that primary electronic device usage exerts both a direct effect on frailty and an indirect effect through nutritional management. Because the study depended on cross-sectional data, the causal relationships among the variables cannot be definitively established, thus emphasizing the need for longitudinal research. Despite this limitation, the findings provide valuable baseline evidence to inform frailty management programs for older adults living alone. Accordingly, future interventions must incorporate strategies aimed at promoting and supporting electronic device usage as a part of comprehensive frailty prevention and management efforts.

## Figures and Tables

**Table 1:** General Characteristics and Study Variables (N = 3423).

Characteristics	Categories	*n* (%) or M (SD)	Range Min–Max		Frailty	
				M ± SD	t or F	*p* Scheffe
Age		75.57 (7.10)	65–98			
Gender	Male	766 (22.4)		0.54 ± 0.87	−8.43	<0.001
	Female ^a^	2657 (77.6)		0.89 ± 1.04		
Education level	≤Elementary school ^a^	1961 (57.3)		1.04 ± 1.07	91.65	<0.001 a > b, c, d
	Middle school ^b^	638 (18.6)		0.58 ± 0.85		
	High school ^c^	692 (20.2)		0.43 ± 0.79		
	≥University ^d^	132 (3.9)		0.39 ± 0.84		
Having a job	Yes	1274 (37.2)		0.57 ± 0.88	10.70	<0.001
	No	2149 (62.8)		0.95 ± 1.06		
Perceived health status	Healthy ^a^	1115 (32.6)		0.38 ± 0.70	419.12	<0.001 a < b < c
	Moderate ^b^	1225 (35.8)		0.63 ± 0.89		
	Unhealthy ^c^	1083 (31.6)		1.45 ± 1.10		
Quality of sleep	Good ^a^	1449 (42.3)		0.52 ± 0.83	200.32	<0.001 a < b < c
	Average ^b^	1214 (35.5)		0.78 ± 0.97		
	Bad ^c^	760 (22.2)		1.39 ± 1.14		
Electronic device usage		3.42 ± 3.41	0–13			
Frailty		0.81 ± 1.01	0–5			
Nutrition management		3.12 ± 3.23	0–10			

M = mean; SD = standard deviation; Min = minimum; Max = maximum.

**Table 2:** Correlations between Study Variables (N = 3423).

Variables	Electronic Device Usage	Nutrition Management	Frailty
	r (*p*)	r (*p*)	r (*p*)
**Electronic device usage**	1		
**Frailty**	−0.34 (<0.001)	0.31 (<0.001)	
**Nutrition management**	−0.17 (<0.001)	1	1

**Table 3:** Mediating effect of nutrition management on the relationship between electronic device usage and frailty (N = 3423).

Step	Independent Variables	Dependent Variables	B	SE	β	t (*p*)	Adj. R^2^	F (*p*)
1	Electronic device usage	Nutrition management	−0.17	0.02	−0.17	−10.23 (<0.001)	0.030	104.82 (<0.001)
2	Electronic device usage	Frailty	−0.10	0.01	−0.33	−20.77 (<0.001)	0.112	431.75 (<0.001)
3	Electronic device usage	Frailty	−0.09	0.01	−0.29	−18.39 (<0.001)	0.180	374.80 (<0.001)
	Nutrition management	Frailty	0.08	0.01	0.26	16.80 (<0.001)		

**Table 4:** Statistical significance of indirect mediation effects (N = 3423).

Effect	Boot SE	95% Confidence Interval	
		Boot LLCI	Boot ULCI
−0.05	0.01	−0.06	−0.04

LLCI = lower limit of confidence interval; ULCI = upper limit of confidence interval.

## Data Availability

The data presented in this study are openly available in MicroData Integrated Service (MDIS) website (https://mdis.mods.go.kr/index.do, accessed on (14 November 2025)).
